# Assessing the performance of a method for case-mix adjustment in the Korean Diagnosis-Related Groups (KDRG) system and its policy implications

**DOI:** 10.1186/s12961-021-00739-5

**Published:** 2021-06-29

**Authors:** Sujeong Kim, Byoongyong Choi, Kyunghee Lee, Sangmin Lee, Sukil Kim

**Affiliations:** 1 Department of Preventive Medicine and Public Health, College of Medicine, The Catholic University, Main building No. 223, 222 Banpodaero, Seoul, Korea; 2 Department of Internal Medicine, Seoul Medical Center, Seoul, Korea; 3 Department of Healthcare Management, Eulji University, Gyeonggi-do, Korea; 4 Department of Community Health Sciences, University of Calgary, Alberta, Canada

**Keywords:** Diagnosis-related groups, Inpatient case mix, Risk adjustment, Prospective payment system

## Abstract

**Background:**

To evaluate the performance of the patient clinical complexity level (PCCL) mechanism, which is the patient-level complexity adjustment factor within the Korean Diagnosis-Related Groups (KDRG) patient classification system, in explaining the variation in resource consumption within age adjacent diagnosis-related groups (AADRGs).

**Methods:**

We used the inpatient claims data from a public hospital in Korea from 1 January 2017 to 30 June 2019, with 18 846 claims and 138 AADRGs. The differences in the total average payment between the four PCCL levels for each AADRG was tested using ANOVA and Duncan’s post hoc test. The three patterns of differences with *R*-squared were as follows: the PCCL reflected the complexity well (valid); the average payment for PCCL 2, 3, and 4 was greater than PCCL 0 (partially valid); the PCCL did not reflect the complexity (not valid).

**Results:**

There were 9 (6.52%), 26 (18.84%), and 103 (74.64%) ADRGs included in the valid, partially valid, and not valid categories, respectively. The average R-squared values were 32.18, 40.81, and 35.41%, respectively, with an average *R*-squared for all patterns of 36.21%.

**Conclusions:**

Adjustment using the PCCL in the KDRG classification system exhibited low performance in explaining the variation in resource consumption within AADRGs. As the KDRG classification system is used for reimbursement under the new DRG-based prospective payment system (PPS) pilot project, with plans for expansion, there should be an overall review of the validity of the complexity and rationality of using the KDRG classification system.

**Supplementary Information:**

The online version contains supplementary material available at 10.1186/s12961-021-00739-5.

## Background

### The importance of complexity adjustment in diagnosis-related groups

Many countries have been concerned about the performance of the diagnosis-related groups (DRG) system for payment accuracy. In the United States, for example, many studies have evaluated whether variables other than the diagnosis variable could be used for the DRG or the predictive performance of cost between various types of case-mix measurement systems [1–7]. Many other countries have adopted and applied other variables such as types of hospitalization/discharge or methodologies that can detect variations within patient groups, taking into account their healthcare context [8–11].

### Healthcare system and payment method in Korea

In Korea, more than 90% of hospitals are privately owned [12], with a more complex patient case mix in private hospitals than public hospitals. The main method for payment is the fee-for-service model, with no separate payments between hospitals and doctors by the National Health Insurance System. The single public insurer (National Health Insurance Service, NHIS) pays 80% of the hospital charge for inpatient stay, and the patient pays the remainder.

There are two types of DRG payment systems for patient classification in Korea that originated from the same patient classification system [13, 14]: (1) the mandatory DRG-based prospective payment system (PPS) for seven diseases, and (2) the new DRG-based PPS for public hospitals. The mandatory DRG-based PPS, including payments for both hospitals and doctors, targets seven relatively simple surgical disease groups, and was first introduced to certain clinics and hospitals in July 2012. It was then extended to all medical institutions in July 2013. Under the pilot project, the new DRG-based PPS targeted public hospitals with physician procedures, expensive therapeutic materials, and some expensive drugs paid separately as fee-for-service payments in the system. Since 2018, the new DRG-based PPS has been extended to private hospitals through voluntary participation.

### Overview of the complexity adjustment of the Korean Diagnosis-Related Groups

The mechanisms for reflecting the complexity of the Korean Diagnosis-Related Groups (KDRG) system (Additional file 1: The Structure of the Korean DRG classification and refinement step of ADRG based on secondary diagnosis), which was developed based on the United States Refined DRG (US R-DRG) and the Australian Refined DRG (AR-DRG), are as follows [15, 16]: (1) the patient's complications and comorbidities (CC) are assigned a severity score based on the CC list; (2) if there are multiple secondary diagnoses, the duplicates are removed by applying an exclusion list; and (3) the disease group severity is adjusted and refined using the patient clinical complexity level (PCCL) calculation formula that calculates the cumulative effects of the multiple diagnoses. The PCCL was designed to prevent similar diseases from being calculated more than once and is intended to reflect the cumulative effect of a patient’s CC [17, 18]. The PCCL value is calculated per patient episode, and the RDRG per age adjacent DRG (AADRG) is determined by considering the statistical criteria and the minimum number of counts [15, 16].

### Follow-up study for previous research

This is a follow-up study of a previous study by Kim et al. [19] that reviewed the validity of the CC severity adjustment mechanism, which is a pre-PCCL calculation step in the KDRG classification system. In the previous study, the validity of the severity adjustment mechanism using CC was reviewed. Our previous study reported that only 114 (19.03%) out of 599 adjacent DRGs (ADRGs) had a valid comorbidities and complications level (CCL) [19]. However, we were not able to evaluate the accuracy of payment at the patient level. As the new DRG-based PPS is extended to private hospitals that have more complex patients than public hospitals in Korea, ensuring accurate payment at the patient level is important. Therefore, this follow-up study aims to validate the accuracy of payment at the patient level using the new DRG-based PPS in a public hospital.

## Methods

### Data

We used inpatient claims data from a general hospital with about 600 beds in Seoul, Korea (1 January 2017 to 30 June 2019). The hospital is a public hospital and one of the reference hospitals (three public and three private medical institutions) whose data have been used to set the base DRG fee for the new DRG-based PPS pilot project. A total of 26 784 claims were available in raw data.

The PCCL score and DRG code per episode were automatically assigned by the DRG grouper distributed by the Health Insurance Review and Assessment Service (HIRA), which is responsible for the development of the KDRG classification system. Basic patient information, diagnoses, and procedures are the mandatory input data for the grouper. To evaluate the validity of the PCCL method, we used the PCCL scores and payment amount based on the fee for service, which is a proxy measurement for cost.

In KDRG, the ADRG is determined by a combination of primary diagnosis and main surgery or procedure that a patient undergoes, and is then classified by age as AADRG. Then, the AADRG is classified as RDRG, reflected by the severity of the secondary diagnoses. In the general DRG classification structure, ADRG is divided into RDRG, but the classification structure in the KDRG follows the order ADRG–AADRG–RDRG. In this paper, AADRGs can be understood as the same concept as the ADRG of the general DRG.

**Fig. 1:**
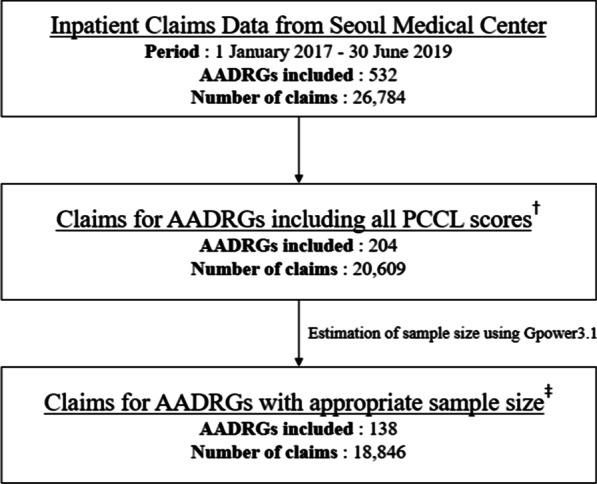
The selection of study data for analysis. *PCCL* patient clinical complexity level, *AADRG* age adjacent DRG. ^†^The PCCL scores consist of 0, 2, 3, and 4 levels. ^‡^The appropriate sample size for analysis of variance (ANOVA) was determined using G*Power according to AADRGs. The sample size means the number of data

A total of 811 AADRGs in the KDRG v1.2 classification were used for the new DRG-based PPS, excluding early death DRG, error DRGs, and pre-major diagnostic category (MDC). Of the 26 784 claims in the general hospital claims database, there were 532 AADRGs (Fig. 1). Only 204 AADRGs in 20 609 claims contained PCCL scores of 0, 2, 3, or 4. We estimated the appropriate number of samples for each AADRG using G*Power 3.1 [20] and chose data on 138 AADRGs in 18 846 claims as analysis data.

### Statistical analyses

The general characteristics of the data are reported as mean ± standard deviation (SD) or as percentage for gender, age, type of insurance, length of stay, and payment amount. We also show data characteristics according to major diagnostic category (MDC) in the KDRG.

Only the AADRGs with adequate sample sizes were selected to report. We used G*Power 3.1 [20] to calculate the minimum sample sizes per AADRG. The alpha was set to 0.05, and the power to 0.8. Effect size was estimated from the SD within each group of each AADRG, the sample size, and mean of log-transformed payment amount from the actual data. For example, in AADRG I6821, where the number of groups = 4 and the SD within each group = 0.2656, the average log-transformed payment amounts were 6.32068, 6.45057, 6.7249, and 7.03847 for sample sizes of 97, 20, 7, and 3, respectively. The estimated effect size was 0.5360507, and the minimum sample size was 44. AADRG I6821 was selected to report because the actual sample size was 127.

The statistical hypothesis was that the average payment amount would increase significantly with an increase in the PCCL score. To evaluate the ability of the PCCL score to explain patient complexity, we performed a one-way analysis of variance (ANOVA) and Duncan’s post hoc test using PCCL scores as an independent variable and the log-transformed payment amount as the dependent variable. The dependent variable is the sum of fees for medical services provided to patients under the fee-for-service payment system (Additional file 2: The diagram of analysis method).

The *R*^2^ value of the ANOVA is presented for the explanatory power of the PCCL on the payment amount.

### Pattern analysis

Based on the same criteria as our previous research, we categorized the results of Duncan’s post hoc test by AADRGs into three different validity patterns: valid, partially valid, and not valid (Additional file 3: Criteria used to classify validity patterns).

The valid pattern included the AADRGs in which the average payment amount increased significantly with increase an in the PCCL score. B6623 in Additional file 4 is a good example. For the partially valid pattern, the average payment amount of PCCL 0 was significantly less than the lowest average payment amount of the other PCCLs. Duncan’s post hoc test for the payment amount of E7202 in Additional file 4 shows that the average payment amounts of PCCL 3 and PCCL 4 were not statistically different from that of PCCL 2, but different from that of PCCL 0. We considered them inappropriate but better than not valid. In the not valid pattern, the average payment amount of PCCL 0 is statistically equal to or greater than the average payment amount of other PCCLs. J6002 in Additional file 4 shows that the average payment amount of PCCL 0 is statistically the same as that of PCCL 2.

## Results

### General characteristics

The number of AADRGs and inpatient claims in the raw and analysis data at the MDC level is shown in Table 1. Of the 532 AADRGs, 138 (25.94%) AADRGs in 18 846 (70.36%) claims were included for analysis.

**Table 1:** The number of age adjacent diagnosis-related groups and claims between raw and analysis data at the major diagnostic characteristic level

MDC	MDC title	No. of AADRGs on raw data, *n* (%)	No. of AADRGs on analysis data, *n* (%)^a^	No. of discharge cases on raw data, *n* (%)	No. of discharge cases on analysis data, *n* (%)^b^
MDC 01	Diseases and disorders of the nervous system	66 (12.41)	14 (21.21)	1 994 (7.44)	1 209 (60.63)
MDC 02	Diseases and disorders of the eye	9 (1.69)	0 (0.0)	615 (2.30)	0 (0.0)
MDC 03	Diseases and disorders of ear, mouth, and throat	38 (7.14)	2 (5.26)	1 011 (3.77)	299 (29.57)
MDC 04	Diseases and disorders of the respiratory system	41 (7.71)	18 (43.90)	4 207 (15.71)	3 404 (80.91)
MDC 05	Diseases and disorders of the circulatory system	38 (7.14)	12 (31.58)	2 353 (8.79)	1 843 (78.33)
MDC 06	Diseases and disorders of the digestive system	58 (10.90)	23 (39.66)	3 862 (14.42)	3 036 (78.61)
MDC 07	Diseases and disorders of the hepatobiliary system and pancreas	28 (5.26)	12 (42.86)	1 646 (6.15)	1 543 (93.74)
MDC 08	Diseases and disorders of the musculoskeletal system and connective tissue	69 (12.97)	20 (28.99)	3 073 (11.47)	2 223 (72.34)
MDC 09	Diseases and disorders of the skin, subcutaneous tissue	25 (4.70)	5 (20.00)	875 (3.27)	220 (25.14)
MDC 10	Endocrine, nutritional, and metabolic diseases and disorders	15 (2.82)	3 (20.00)	963 (3.60)	809 (84.01)
MDC 11	Diseases and disorders of the kidney and urinary tract	32 (6.02)	15 (46.88)	1 893 (7.07)	1 720 (90.86)
MDC 12	Diseases and disorders of male reproductive system	14 (2.63)	1 (7.14)	286 (1.07)	49 (17.13)
MDC 13	Diseases and disorders of the female reproductive system	23 (4.32)	3 (13.04)	629 (2.35)	192 (30.52)
MDC 14	Pregnancy, childbirth, and puerperium	14 (2.63)	1 (7.14)	722 (2.70)	303 (41.97)
MDC 16	Diseases and disorders of the blood and blood-forming organs and immunological disorders	4 (0.75)	2 (50.00)	244 (0.91)	232 (95.08)
MDC 17	Neoplastic disorders (haematological and solid neoplasms)	9 (1.69)	2 (22.22)	1 586 (5.92)	1 530 (96.47)
MDC 18–2	Infectious and parasitic diseases	16 (3.01)	1 (6.25)	158 (0.59)	26 (16.46)
MDC 19	Mental diseases and disorders	16 (3.01)	2 (12.50)	376 (1.40)	81 (21.54)
MDC 20	Alcohol/drug use and alcohol/drug-induced organic mental disorders	1 (0.19)	0 (0.0)	42 (0.16)	0 (0.0)
MDC 21–2	Injuries, poisoning, and toxic effects of drugs	14 (2.63)	2 (12.49)	242 (0.90)	127 (54.48)
MDC 22	Burns	2 (0.38)	0 (0.0)	7 (0.03)	0 (0.0)
Total		532 (100)	138 (25.94)	26 784 (100)	18 846 (70.36)

*MDC* major diagnostic category, *AADRG* age adjacent diagnosis-related groups

^a^The denominator of the ratio is the number of AADRGs on raw data

^b^The denominator of the ratio is the number of discharge cases on raw data

### The validity pattern analysis

A summary of the pattern analysis of the validity of the PCCL scores is shown in Table 2. In nine AADRGs (6.52%), the average payment amount increased significantly with an increase in the four PCCL scores (0, 2, 3, 4), indicating a valid pattern. There were 26 AADRGs (18.84%) that were partially valid or had an average payment amount of PCCL 0 that was significantly less than the lowest average amount of other PCCL scores, and there were 103 AADRGs that were not valid (74.64%), meaning that they did not reflect the complexity between average payment and PCCL score, suggesting that the average amount of PCCL 0 was not significantly different from that of other PCCLs.

**Table 2:** The results of validity pattern analysis

Validity pattern	Total *N* (%)	*R*-Squared^a^ (%)
Valid	9 (6.52)	32.18
Partially valid	26 (18.84)	40.81
Not valid	103 (74.64)	35.41
Total (*n*)	138 (100)	36.21

^a^The *R*^2^ of AADRGs belonging to each pattern group were counted on average

If we consider the valid and partially valid patterns as acceptable results in the current four PCCL scores reflecting the variation in average payment amount within AADRGs, the average payment amount of 103 AADRGs (74.64%) is not accounted for by the current four PCCL scores. On the other hand, if we consider only the valid pattern as an acceptable result, the average payment for 129 AADRGs (94.5%) is not accounted for by the four PCCL scores. The average *R*^2^ for the payment amount of AADRGs by the four PCCL scores in the valid, partially valid, and not valid patterns is 36.21%. The average *R*^2^ of the valid pattern between the average payment amount of AARDGs per PCCL score is 32.18%, which is lower than the average *R*^2^ of the partially valid or not valid patterns.

## Discussion

This is the first study to evaluate the mechanism of patient-level complexity adjustment in the KDRG. Our results showed that using PCCL for the new DRG-based PPS exhibited low performance. A study conducted in Australia reported a newly developed complexity adjustment mechanism, since the existing PCCL measure developed using limited data on length of stay had not been revised since it was first introduced [18]. Similarly to our study, the Australian study also reported poor performance using the PCCL complexity adjustment on their hospital cost data.

The low performance of the PCCL adjustment in determining average payments using the KDRG may potentially be due to the various factors used to calculate PCCLs, such as the CC list, CCLs [15], and CC exclusion list [21], which have not been updated since their introduction, as stated in the our previous study [19]. Another reason for the poor performance of the PCCL adjustment may be inaccuracy in secondary diagnosis coding [22]. The current coding guideline used in Korea is based on the guidelines used by other countries for statistical purposes to determine the prevalence and mortality of disease and not for DRG-based payments [23]. It is currently revised and issued by the National Statistical Office under the Ministry of Economy and Finance, not by the Ministry of Health and Welfare. This administrative structure makes it difficult to reflect clinical reality in various healthcare fields in the guideline.

### Limitations

There are limitations to this study. The results of the study may not be generalizable to the total patient population covered by the new DRG-based PPS or may not represent all of the AADRGs in the KDRG because of the small sample collected from a single hospital*.* Our research showed poor performance of the complexity adjustment mechanism in the KDRG system, despite the fact that the hospital that conducted this research has a greater proportion of patients with more common and moderate-complexity diseases than tertiary hospitals. This suggests that the performance may be worse in hospitals with a more complex patient case mix. Access to the claims data related to the new DRG-based PPS pilot project is currently limited to the public. Further studies should be done for validation of the whole AADRG using a sufficient quantity of claims data.

Furthermore, not all the AADRGs were evaluated, as we were limited to the number of DRGs found in our inpatient claims database. We may have overestimated AADRGs with not valid pattern analysis. However, we tried to ensure the statistical power by including the AADRGs which had sample sizes greater than or equal to the minimum size calculated by G*Power.

Lastly, we assessed the validity of the PCCL adjustment with the KDRG system on the medical charges and not the cost [24]. The fee-for-service charge was set up including payments for hospitals and doctors under government control and was used as a proxy to identify resource consumption. Because there are no cost data for medical services in Korea, we are not able to suggest data for them. The government is planning to collect cost data from the hospitals that participated in the new KDRG project.

### Significance

In most countries, DRG is used mainly as a basis for budget allocation to increase the transparency and efficiency of medical services [25]. In Korea, however, under the existing fee-based payment system, DRG was introduced to expand health insurance coverage by controlling uninsured medical services and to contain the rapidly increasing trend in national medical expenditure. The predetermined DRG fee for each disease group is paid directly to healthcare providers for their services. As of April 2020, there were 52 private hospitals participating in the new DRG-based PPS pilot project, with the government providing up to 30% policy participation incentives to hospitals. According to hospitals that have participated in the pilot project, the hospitals have negative revenue after excluding participation incentives [26, 27]. This indicates that the compensation for the provision of medical services based on the PCCL adjustment in the KDRG classification system does not cover the true medical costs. By 2022, however, participation incentives for the new DRG-based PPS are expected to decrease. Thus, hospitals will be reimbursed for inpatient services solely on the DRG-specific fees calculated based on the cost currently being collected by the government.

The most accurate and appropriate compensation using the new DRG-based PPS can be determined with a stable patient classification system, a reasonable complexity adjustment mechanism, and cost data.

The severity adjustment mechanism in the DRG contributes to ensuring homogeneity within disease groups, and it is especially important to improve the accuracy of payment when it used as a payment unit. Experts argue for quickly replacing the fee-for-service system with the new DRG-based PPS to stabilize the rapid increase in national medical expenditure and to increase health insurance coverage [28]. Considering the expansion of the application of the new DRG-based PPS pilot project to 200 medical institutions including private hospitals by 2022, it is importance to ensure payment accuracy using the new DRG-based PPS by evaluating whether the PCCL mechanism for adjusting the patient-level complexity is functioning properly in the KDRG [29]. Therefore, we need to prepare for the financial problems of the Korean healthcare system that may be caused by improper classification when introducing a new payment system.

## Conclusion

The poor performance of PCCLs, a mechanism for patient-level complexity adjustment, in the KDRG system suggests that there should be an overall review of the validity and rationality of using the PCCLs in the KDRG classification system for reimbursement.

Although changes in the payment mechanism for providers is inevitable, stabilization and rationality of the system’s components must be ensured, as the payment system is a factor that can affect the providers, insurers, and ultimately the patients. Therefore, when designing systems and implementing policies, policy-makers should take a more cautious approach considering their long-term impact.

## Supplementary Information

**Additional file 1.** The Structure of the Korean DRG classification and Refinement steps of ADRG based on secondary diagnosis.

**Additional file 2.** The diagram of analysis method.

**Additional file 3.** Criteria used to classify validity patterns.

**Additional file 4.** Examples of validity pattern analysis.

## Data Availability

The datasets generated during and/or analysed during the current study are not publicly available due to the nature of the data owned by the medical institution but are available from the corresponding author on reasonable request.

## Abbreviations

DRG: Diagnosis-related groups
PPS: Prospective payment system
KDRG: Korean Diagnosis-Related Groups
US R-DRG: United States Refined Diagnosis-Related Groups
AR-DRG: Australian Refined Diagnosis-Related Groups
CC: Complications and comorbidities
PCCL: Patient clinical complexity level
RDRG: Refined diagnosis-related group
AADRG: Age adjacent diagnosis-related group
CCL: Comorbidities and complications level
HIRA: Health Insurance Review and Assessment Service
ADRG: Adjacent diagnosis-related group
MDC: Major diagnostic category
ANOVA: Analysis of variance
