# Supplementary material for: Assessing the performance of a method for case-mix adjustment in the Korean Diagnosis-Related Groups (KDRG) system and its policy implications
Source: Health Res Policy Syst. 2021 Jun 29;19:98. doi: 10.1186/s12961-021-00739-5 (PMC8243480; doi:10.1186/s12961-021-00739-5)
Supplement: Supplementary file 1 — Additional file 1. The Structure of the Korean DRG classification and Refinement steps of ADRG based on secondary diagnosis. [file 12961_2021_739_MOESM1_ESM.docx]

**Additional file 1.**

The Structure of Korean DRG classification


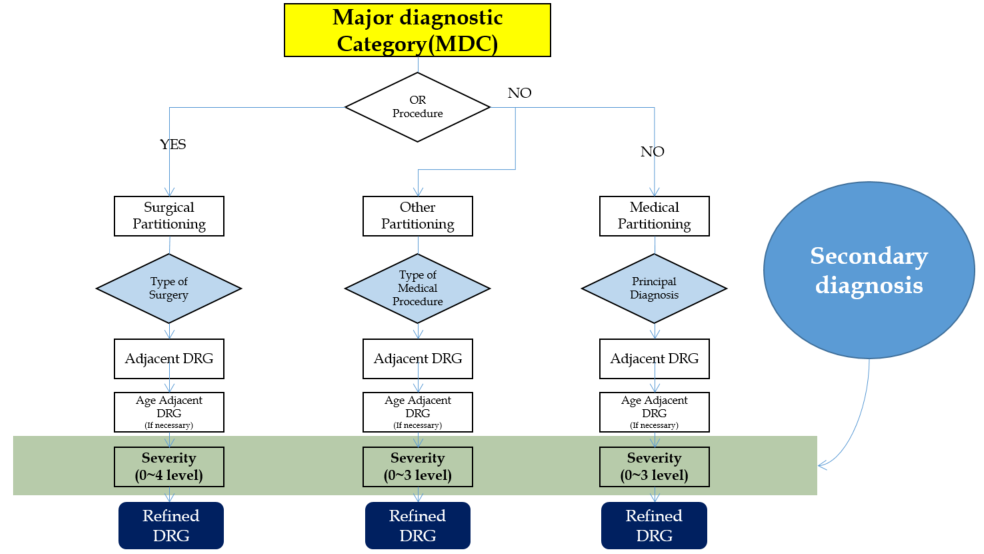


The Refinement step of ADRG based on secondary diagnosis


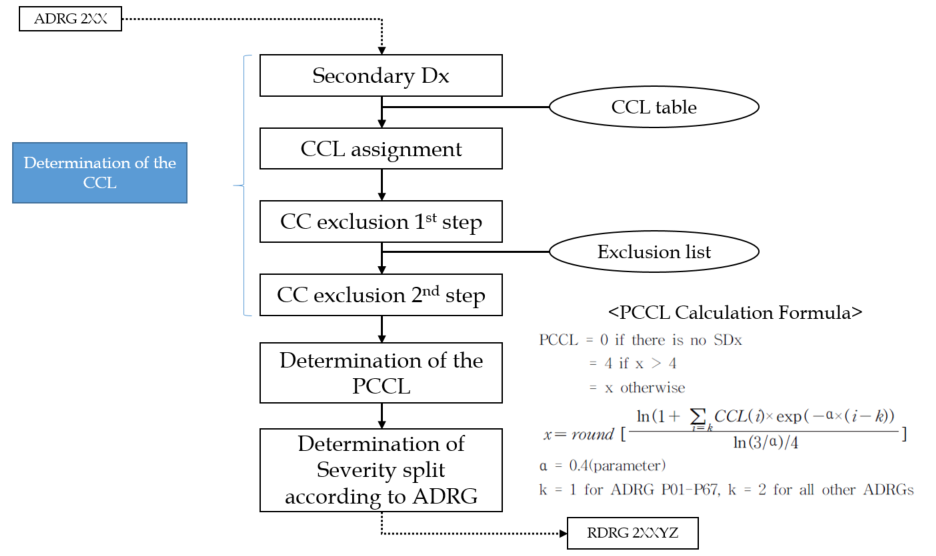


Dx: Diagnosis; CCL: Complication and Comorbidity level; CC: Complication and Comorbidity; PCCL: Patient Clinical Complexity Level;
