# Supplementary material for: Assessing the performance of a method for case-mix adjustment in the Korean Diagnosis-Related Groups (KDRG) system and its policy implications
Source: Health Res Policy Syst. 2021 Jun 29;19:98. doi: 10.1186/s12961-021-00739-5 (PMC8243480; doi:10.1186/s12961-021-00739-5)
Supplement: Supplementary file 2 — Additional file 2. The diagram of analysis method. [file 12961_2021_739_MOESM2_ESM.docx]

**Additional file 2.**

The diagram of analysis method


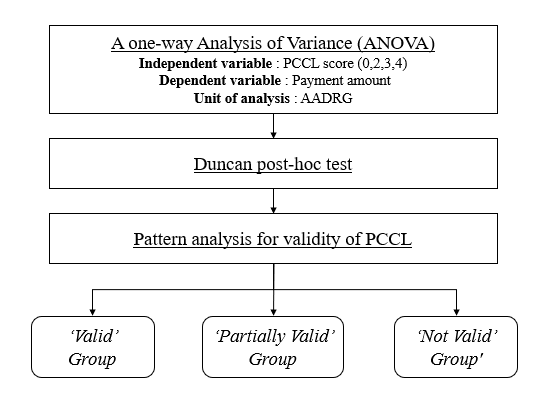


PCCL: Patient Clinical Complexity Level; AADRG: Age Adjacent DRG;
