# Supplementary material for: Assessing the performance of a method for case-mix adjustment in the Korean Diagnosis-Related Groups (KDRG) system and its policy implications
Source: Health Res Policy Syst. 2021 Jun 29;19:98. doi: 10.1186/s12961-021-00739-5 (PMC8243480; doi:10.1186/s12961-021-00739-5)
Supplement: Supplementary file 3 — Additional file 3. Criteria used to classify validity patterns. [file 12961_2021_739_MOESM3_ESM.docx]

**Additional file 3.**

Criteria used to classify validity patterns

| Criteria | Validity pattern | | |
| --- | --- | --- | --- |
|  | Valid | Partially Valid | Not Valid |
| The Average amount of PCCL 0 is significantly different from those of other PCCLs | Yes | Yes | No |
| The order of average amount by PCCLs is kept | Yes | No | No |

PCCL: Patient Clinical Complexity Level;
