# Supplementary material for: Assessing the performance of a method for case-mix adjustment in the Korean Diagnosis-Related Groups (KDRG) system and its policy implications
Source: Health Res Policy Syst. 2021 Jun 29;19:98. doi: 10.1186/s12961-021-00739-5 (PMC8243480; doi:10.1186/s12961-021-00739-5)
Supplement: Supplementary file 4 — Additional file 4. Examples of validity pattern analysis. [file 12961_2021_739_MOESM4_ESM.docx]

**Additional file 4.**

Examples of validity pattern analysis

| PCCL value | Average payment amount (KRW) | N | Duncan Grouping^†^ | | | |
| --- | --- | --- | --- | --- | --- | --- |
|  |  |  | A | B | C | D |
| **Valid**  KDRG B6623 Non-Hemorrhagic Stroke, Age > 69 | | | | | | |
| 4 | 10,140,966 | 199 |  |  |  |  |
| 3 | 7,919,015 | 63 |  |  |  |  |
| 2 | 5,247,285 | 96 |  |  |  |  |
| 0 | 3,993,630 | 43 |  |  |  |  |
| **Partially valid**  KDRG E7202 Other Respiratory System Diagnoses, Age >64 | | | | | | |
| 4 | 5,105,493 | 9 |  |  |  |  |
| 3 | 3,313,825 | 20 |  |  |  |  |
| 2 | 3,545,162 | 6 |  |  |  |  |
| 0 | 2,041,996 | 38 |  |  |  |  |
| **Not valid** | | | | | | |
| KDRG J6002 Skin Ulcers, Age >64 | | | | | | |
| 4 | 12,426,251 | 9 |  |  |  |  |
| 3 | 8,913,230 | 11 |  |  |  |  |
| 2 | 7,889,448 | 10 |  |  |  |  |
| 0 | 5,457,742 | 10 |  |  |  |  |

PCCL: Patient Clinical Complexity Level; KDRG: Korean Diagnosis-related Groups; KRW: Korean Won (1 US Dollar = 1215 Korean Won as of April 14, 2020);

;

^†^The grey cell means valid, the black cell not valid.
